# Metabolic Energy Contributions During High-Intensity Hatha Yoga and Physiological Comparisons Between Active and Passive (*Savasana*) Recovery

**DOI:** 10.3389/fphys.2021.743859

**Published:** 2021-09-24

**Authors:** Kwang-Ho Lee, Hyo-Myeong Ju, Woo-Hwi Yang

**Affiliations:** Graduate School of Sports Medicine, CHA University, Seongnam, South Korea

**Keywords:** energy demands, phosphagen system, glycolytic system, oxidative system, blood lactate, resynthesis

## Abstract

**Purpose:** The objective of this study was to investigate metabolic energy contributions during high-intensity hatha yoga (HIHY) and to compare changes in physiological variables between active and passive recovery methods.

**Methods:** The study involved 20 women yoga instructors (*n* = 20) who performed 10 min of HIHY (vigorous sun salutation). Upon completion, they were randomly assigned to either active (walking; *n* = 10) or passive (*savasana*; *n* = 10) recovery groups for a period of 10 min. During HIHY, physiological variables such as heart rate (HR_peak_ and HR_mean_), oxygen uptake (VO_2peak_ and VO_2mean_), and blood lactate concentrations (peak La^−^) were measured. Energetic contributions (phosphagen; W_PCR_, glycolytic; W_Gly_, and oxidative; W_Oxi_) in kJ and % were estimated using VO_2_ and La^−^ data. Furthermore, the metabolic equivalents (METs) of VO_2peak_ and VO_2mean_ were calculated. To compare different recovery modes, HR_post_, ΔHR, VO_2post_, ΔVO_2_, recovery La^−^, and recovery ΔLa^−^ were analyzed.

**Results:** The results revealed that HR_peak_, VO_2peak_, and peak La^−^ during HIHY showed no differences between the two groups (*p* > 0.05). Values of HR_peak_, HR_mean_, METs of VO_2peak_ and VO_2mean_, and La^−^ during HIHY were 95.6% of HR_max_, 88.7% of HR_max_, 10.54 ± 1.18, 8.67 ±.98 METs, and 8.31 ± 2.18 mmol·L^−1^, respectively. Furthermore, W_Oxi_ was significantly higher compared with W_PCR_, W_Gly_, and anaerobic contribution (W_PCR_ + W_Gly_), in kJ and % (*p* < 0.0001). VO_2post_ and recovery ΔLa^−^ were significantly higher in the active recovery group (*p* < 0.0001, *p* = 0.0369, respectively). Values of ΔVO_2_ and recovery La^−^ were significantly lower in the active group compared with the passive group (*p* = 0.0115, *p* = 0.0291, respectively).

**Conclusions:** The study concluded that high-intensity hatha yoga which was performed for 10 min is a suitable option for relatively healthy people in the modern workplace who may have hatha yoga experience but do not have time to perform a prolonged exercise. Following active recovery, they can participate in further HIHY sessions during short breaks. Furthermore, a faster return to work can be supported by physiological recovery.

## Introduction

The greatest public health problem of the 21st century is physical inactivity which is usually the consequence of modern sedentary lifestyles (Booth et al., 2000; Trost et al., 2014). Most international guidelines for physical activity recommend at least 150 min of moderate-intensity physical activity (3–5.9 metabolic equivalents; METs) or 75 min of vigorous-intensity aerobic physical activity (≥6 METs) per week for adults (Ainsworth et al., 2011; Hallal et al., 2012; Brinsley et al., 2021). However, estimates based on self-reported data show that 40–60% of the general adult population are not sufficiently active (Hallal et al., 2012). This may lead to non-communicable diseases, including cardiovascular, coronary heart disease, diabetes, and cancer, which account for seven of the ten most common worldwide reasons for premature death (Hallal et al., 2012; Brinsley et al., 2021).

The modern workplace has recently been recognized as an alternative setting for physical activity or exercise for people who may not have time, e.g., during lunch break, to participate in more formal exercise sessions (Kuoppala et al., 2008; Dalager et al., 2016). In this regard, hatha yoga (HY) lends itself to forming part of a general health regimen to prevent physical inactivity (Larson-Meyer, 2016). Additionally, HY aims to improve the body, breath, and mind and prepare self-realization as an alternative form of exercise (Schmalzl et al., 2015; Papp et al., 2019). Previous review and meta-analytic findings have shown that HY decreases blood pressure, blood lipids, glycosylated hemoglobin, low-density lipoprotein, and increases high-density lipoprotein cholesterol (Hagins et al., 2013; Cramer et al., 2014).

However, the common HY program lasts for approximately an hour, which is unsuitable for most people in the workplace. Therefore, a program of high-intensity interval training (HIIT) is an alternative with preferred physical exercises which are also ranked in the top 10 fitness trends of the American College of Sports Medicine (Thompson, 2021). HIIT improves cardiovascular fitness as measured by maximal oxygen uptake (VO_2max_) and includes repeated rounds of exercise that achieve >90% of maximal heart rate (HR_max_), the second ventilatory threshold (>VT_2_), over second lactate threshold (>4 mmol·L^−1^; zone 3: high-intensity exercise), and >85% of peak and maximal oxygen uptake (VO_2peak_ and VO_2max_) (Billat, 2001; Treff et al., 2019; Jamnick et al., 2020).

Hatha yoga is considered as a low-to-moderate-intensity physical activity based on MET values and percentages of HR_max_ and VO_2max_ (Hagins et al., 2007; Ainsworth et al., 2011; Ray et al., 2011). Furthermore, HY can be a form of high-intensity exercise (HIE) (Papp et al., 2019). High-intensity hatha yoga (HIHY) includes vigorous sun salutation (SS) physical exercises (*asanas*) at rapid speed. The most common exercise sequence of HY programs consists of SS (Pascoe and Bauer, 2015; Larson-Meyer, 2016; Papp et al., 2019).

Typically, meditative relaxation (*savasana*) such as passive recovery is conducted after HY and HIHY (Sharma et al., 2007; Papp et al., 2019). However, this recovery method is not suitable after HIHY in the workplace. In light of this, active recovery helps regenerate metabolic pathways which provide greater oxygen uptake (VO_2_) and O_2_ transfer into muscle cells, both of which are necessary for the resynthesis of adenosine triphosphate (ATP) (Menzies et al., 2010; Cupeiro et al., 2016; Yang et al., 2020). The lactate shuttle mechanism plays a crucial role in lactate clearance (Brooks, 2018). Lactate links glycolytic and oxidative energy systems. During active recovery, the accumulated lactate is predominantly re-metabolized by the cell-cell lactate shuttle, and by the Cori cycle and gluconeogenesis. These mechanisms are supported by increased hepatic blood flow during the active recovery phase (Nielsen et al., 1999; Yang et al., 2020). Furthermore, active recovery (low-intensity) activates key enzymes and hormonal regulators of gluconeogenesis such as phosphofructokinase, pyruvate carboxylase, phosphoenolpyruvate carboxykinase, glucagon, cortisol, and other related regulators (Yang et al., 2020).

At present, it is unclear how different energy systems contribute during HIHY. In general, yoga studies have focused on the psychological aspects and benefits, and during HIHY only physiological parameters such as VO_2peak_, peak lactate concentration (peak La^−^), and peak heart rate (HR_peak_) have been analyzed. Following HIHY, the traditional passive recovery process of *savasana* has commonly been utilized although an active recovery causes faster physiological regeneration. Therefore, this study aimed to define the different energetic contributions during HIHY and to compare the magnitude of changes in physiological parameters between passive and active recovery after HIHY.

## Materials and Methods

### Ethical Approval

This study was approved by the Institutional Ethics Committee of CHA University (No. 1044308-202007-HR-026-02). The applied protocols align with the Declaration of Helsinki. All participants signed an informed consent form.

### Participants

In this study, 20 female yoga instructors (*n* = 20) participated. They were recruited from Korea Yoga Alliance (KYA) in the Seoul region and had completed the yoga teacher 300-h program (RYT 300) before study participation. All participants practiced yoga for at least more than 5 years. They practiced yoga independently for 10–12 h per week, without performing any other exercise. The anthropometric parameters of all participants were as follows (*M* ± *SD*): age: 31.0 ± 4.2 years, height: 163.7 ± 4.2 cm, bodyweight: 54.6 ± 5.3 kg, body fat: 24.1 ± 5.4%, BMI: 20.4 ± 1.9 kg·m^−2^ (active recovery group (*n* = 10); age: 28.7 ± 4.4 years, height: 162.4 ± 3.5 cm, bodyweight: 53.9 ± 4.1 kg, body fat: 26 ± 4.6%, BMI: 20.4 ± 1.2 kg·m^−2^, passive recovery group (*n* = 10); age: 33.3 ± 2.7 years, height: 165 ± 4.5 cm, bodyweight: 55.4 ± 6.5 kg, body fat: 22.6 ± 5.8%, BMI: 20.3 ± 2.5 kg·m^−2^) (Table 1). After lunchtime, participants rested for 2 h and conducted the HIHY experiment. The participants did not take any medication during the test procedures and abstained from alcohol and nicotine for at least 24 h before the experiment.

**Table 1:** Anthropometric data.

**Parameters**	**Active recovery (*n* = 10)**	**Passive recovery (*n* = 10)**	**All participants (*n* = 20)**
	**(Mean ± SD)**	**(Mean ± SD)**	**(Mean ± SD)**
Age (years)	28.70 ± 4.45	33.30 ± 2.71	31.00 ± 4.29
Height (cm)	162.41 ± 3.56	165.09 ± 4.58	163.75 ± 4.22
Body weight (kg)	53.94 ± 4.18	55.40 ± 6.51	54.67 ± 5.37
Body fat (%)	26.08 ± 4.68	22.63 ± 5.80	24.18 ± 5.43
BMI (kg·m^−2^)	20.44 ± 1.26	20.35 ± 2.53	20.40 ± 1.95

### Experimental Design

**Figure 1:**
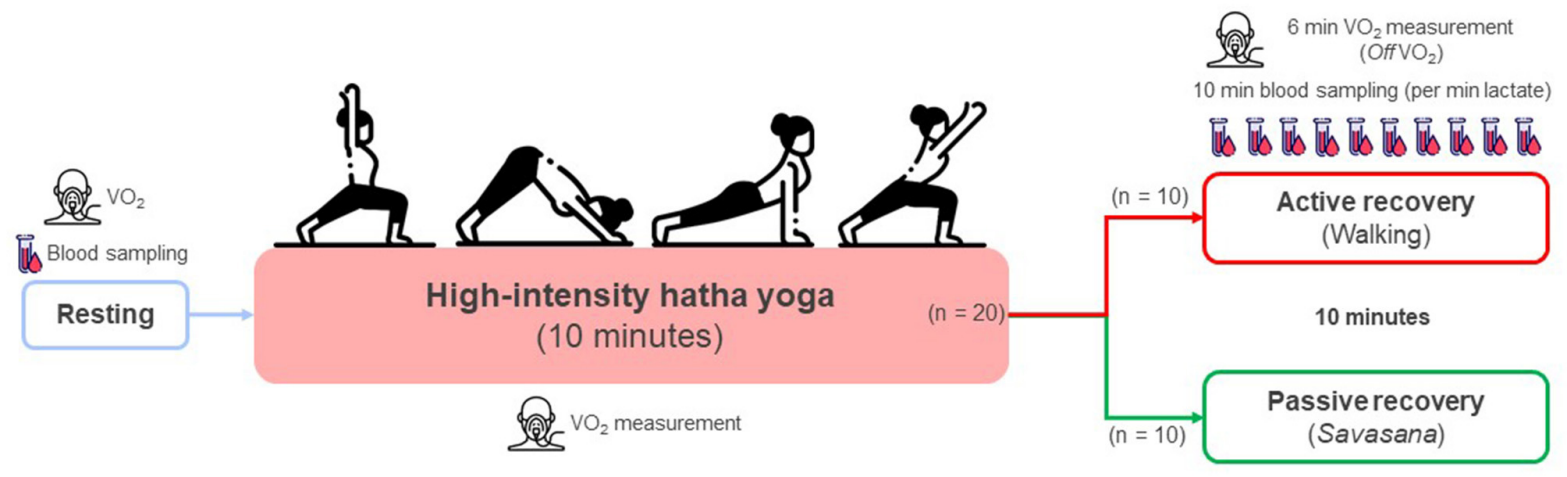
Study procedure. All subjects conducted high-intensity hatha yoga (*n* = 20) and were randomly separated into active (walking; *n* = 10) and passive (*savasana*; *n* = 10) recovery groups.

**Figure 2:**
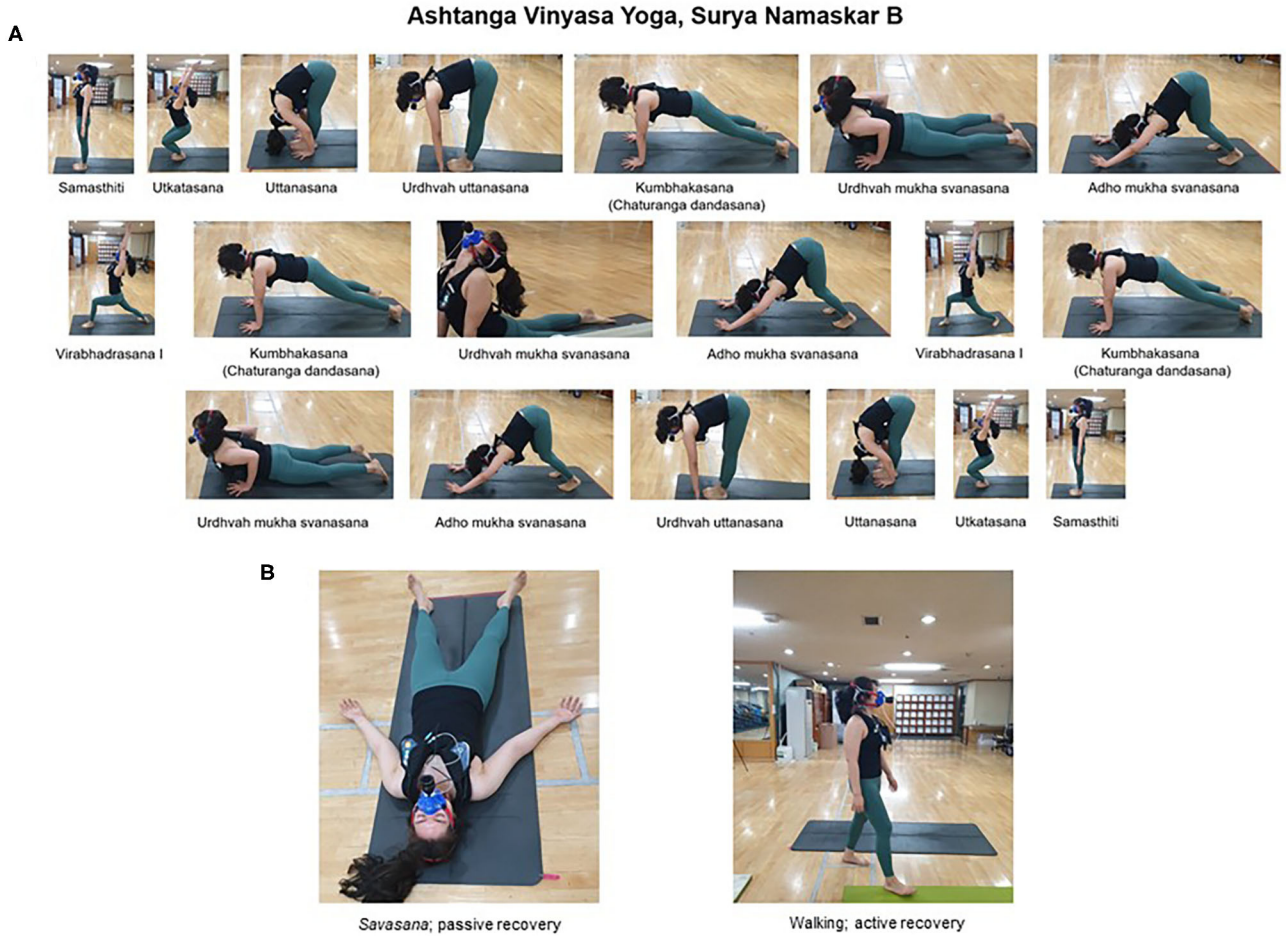
**(A)** Sequence of high-intensity hatha yoga: Ashtanga Vinyasa Yoga, Surya Namaskar B (10-min duration). **(B)** *Savasana*; passive recovery, Walking; active recovery (40–45% of estimated maximal heart rate, 10-min duration).

All participants conducted HIHY (*n* = 20) and were randomly separated into active (walking; *n* = 10) and passive (*savasana*; *n* = 10) recovery groups (Figure 1). HIHY consisted of 19 SS physical exercises (*asanas*) of the Surya Namaskar B sequence (Figure 2A). The HIHY duration of each movement lasted 1.5 s using a metronome and the entire HIHY was conducted for 10 min, which was modified from a previous study (Potiaumpai et al., 2017). Active recovery was performed by walking while maintaining 40–45% of the estimated maximal heart rate (Gellish et al., 2007; Guru et al., 2013) while the passive recovery was conducted in the lying position for 10 min (Figure 2B) (Sharma et al., 2007).

### Anthropometry, Blood Sampling, and Processing

Anthropometric parameters were assessed and measured using 8-electrode segmental multi-frequency (20–100 kHz) bioelectrical impedance analysis (BIA) (InBody 270; InBody Co. Ltd., Seoul, Korea) which enables segmental impedance measurement of arms and legs. The maximal heart rate was estimated using an equation described in the previous study (Gellish et al., 2007). In addition, METs of VO_2peak_ and VO_2mean_ during HIHY were calculated (Ainsworth et al., 2011). During 5 min rest, 10 min HIHY, and 10 min recovery phase, monitoring of heart rate using a Polar H10 (Polar Electro, Kempele, Finland) (HR_peak_, HR_mean_, HR_post_, and ΔHR), oxygen uptake (VO_2peak_, VO_2mean_, VO_2post_, and ΔVO_2_), and blood lactate concentration (peak La^−^, recovery La^−^, and recovery ΔLa^−^) was performed. Capillary blood (20 μL) was sampled from the earlobe before and after HIHY as well as from the 1st to the 10th minute after different recovery to measure blood lactate concentration. La^−^ was analyzed by an enzymatic-amperometric sensor chip system (Biosen C-line, EKF diagnostics sales, GmbH, Barleben, Germany). Oxygen uptake was measured breath-by-breath using a mobile gas analyzer MetaMax 3B (Cortex Biophysik, Leipzig, Germany). The gas analyzer was calibrated using calibration gas (15% O_2_, 5% CO_2_; Cortex Biophysik, Leipzig, Germany), and the turbine volume transducer was calibrated with a 3 L syringe (Hans Rudolph, Kansas City, MO, USA).

### Calculations of Metabolic Energy Contribution

Calculations of energetic contribution were based on measurement of VO_2_ during HIHY and peak La^−^, and VO_2_ after HIHY, respectively (Campos et al., 2012). The phosphagen system contribution (W_PCR_) was calculated by considering the fast component of excess VO_2_ after HIHY (EPOC_FAST_; 6 min *Off* VO_2_ kinetics). The value of W_PCR_ was estimated by subtracting rest VO_2_ (VO_2rest_) from the fast component VO_2post_. The VO_2post_ data were fitted to a mono-exponential model because the slow component of the bi-exponential model was negligible (de Campos Mello et al., 2009; Campos et al., 2012). The contribution of the glycolytic system (W_Gly_) was calculated as La^−^ after HIHY, assuming that the accumulation of 1 mmol·L^−1^ is equivalent to 3 mL O_2_ kg^−1^ of body mass (di Prampero and Ferretti, 1999). The difference in La^−^ (ΔLa^−^) was calculated as the lactate concentration after HIHY, minus the lactate concentration at rest. The oxidative energy (W_Oxi_) was estimated by subtracting VO_2rest_ from VO_2_ during HIHY by the trapezoidal method in which areas under the curve were divided into sections and then the sum of each trapezoid was used to estimate the integral (Campos et al., 2012; Yang et al., 2018; Park et al., 2021). The value of VO_2rest_ was determined in the standing position from the average of the last 30 s of a 5 min period (Campos et al., 2012). The caloric quotient of 20.92 kJ was utilized in all three energy systems (Gastin, 2001). The total energy demand was estimated as the sum of the three energy systems (W_PCR_ + W_Gly_ + W_Oxi_) (di Prampero and Ferretti, 1999; de Campos Mello et al., 2009; Campos et al., 2012; Yang et al., 2018; Park et al., 2021).

### Statistical Analyses

All data were statistically analyzed using GraphPad Prism 9.1.2 (GraphPad Prism Software, La Jolla, CA, USA). The data are presented as *M* ± *SD* and normal distribution was performed using the Shapiro-Wilk test. Energetic contribution variables (kJ and %) were compared using a repeated-measures ANOVA with Bonferroni *post-hoc* testing. Other physiological variables were analyzed by independent *t*-test and Mann-Whitney-U rank test. The effect sizes (Cohen's *d* and *Z*/√*N*) were calculated and thresholds for small, moderate, and large effects were 0.2, 0.5, and 0.8 (parametric), and 0.1, 0.3, and 0.5 (non-parametric), respectively (Fritz et al., 2012). Statistical difference was considered significant at *p* < 0.05 and *p* < 0.01.

## Results

### Physiological Parameters and Energetic Contribution During HIHY

Physiological parameters showed no significant differences between active and passive groups during HIHY (Tables 2, 3). Values of HR_peak_, HR_mean_, METs of VO_2peak_ and VO_2mean_, and La^−^ during HIHY were 95.6% of HR_max_, 88.7% of HR_max_, 10.54 ± 1.18, 8.67 ± 0.98 METs, and 8.31 ± 2.18 mmol·L^−1^, respectively (Table 2).

**Table 2:** Energetic contribution and physiological parameters during high-intensity hatha yoga.

**Parameters**	**Participants (*n* = 20)**	**Significance**	**Effect size (ES)**
	**(Mean ± SD)**	**(*p*)**	**(*d*)**
W_PCR_ (kJ)	33.95 ± 11.32	0.0232* vs. W_Gly_ (kJ)	*d* = 0.93
W_Gly_ (kJ)	24.90 ± 7.63	<0.0001**** vs. W_Oxi_ (kJ)	*d* = −1.30
Anaerobic (kJ) (W_PCR_ + W_Gly_)	58.84 ± 14.25	<0.0001**** vs. W_Oxi_ (kJ)	*d* = 7.41
W_Oxi_ (kJ)	279.33 ± 47.93	<0.0001**** vs. W_PCR_ (kJ)	*d* = 7.04
W_Total_ (kJ)	333.68 ± 47.47		
W_PCR_ (%)	10.34 ± 3.69	0.0437* vs. W_Gly_ (%)	*d* = 0.79
W_Gly_ (%)	7.70 ± 2.88	<0.0001**** vs. W_Oxi_ (kJ)	*d* = −17.35
Anaerobic (%) (W_PCR_ + W_Gly_)	18.04 ± 5.32	<0.0001**** vs. W_Oxi_ (kJ)	*d* = 12.01
W_Oxi_ (%)	81.96 ± 5.32	<0.0001**** vs. W_PCR_ (kJ)	*d* = 15.64
Estimated HR_max_ (beats·min^−1^)	185.30 ± 3.00		
HR_peak_ (beats·min^−1^)	177.21 ± 11.77		
HR_mean_ (beats·min^−1^)	164.47 ± 12.14		
VO_2peak_ (mL·kg^−1^·min^−1^)	36.90 ± 4.14		
METs (VO_2peak_)	10.54 ± 1.18		
VO_2mean_ (mL·kg^−1^·min^−1^)	30.35 ± 3.44		
METs (VO_2mean_)	8.67 ± 0.98		
Peak La^−^ (mmol·L^−1^)	8.31 ± 2.18		

*W_PCR_, W_Gly_, W_Oxi_, absolute (kJ) and relative (%) energetic contribution from phosphagen, glycolytic, and oxidative system; HR_max_, estimated maximal heart rate; HR_peak_, highest heart rate; HR_mean_, mean heart rate; peak La^−^, highest level of blood lactate; METs, metabolic equivalents; VO_2peak_, highest oxygen uptake; VO_2mean_, mean oxygen uptake; anaerobic, phosphagen + glycolytic energy contributions*.

^*^p < 0.05, ^****^p < 0.0001.

**Table 3:** Physiological parameters during high-intensity hatha yoga and 10 min recovery between different groups.

**Parameters**	**Active recovery (*n* = 10)**	**Passive recovery (*n* = 10)**	**Significance**	**Effect size (ES)**
	**(Mean ± SD)**	**(Mean ± SD)**	**(*p*)**	**(*d* and *r*)**
HR_peak_ (beats·min^−1^)	175.77 ± 9.34	178.65 ± 13.09	ns	
HR_post_ (beats·min^−1^)	116.87 ± 9.88	107.97 ± 11.66	ns	
ΔHR (beats·min^−1^)	58.91 ± 12.41	70.68 ± 15.85	ns	
VO_2peak_ (mL·kg^−1^·min^−1^)	38.21 ± 3.60	35.59 ± 4.40	ns	
VO_2post_ (mL·kg^−1^·min^−1^)	12.80 ± 1.40	5.77 ± 1.65	<0.0001****	*r* = −0.84
ΔVO_2_ (mL·kg^−1^·min^−1^)	25.41 ± 3.89	29.82 ± 2.91	0.0115*	*r* = −0.45
Peak La^−^ (mmol·L^−1^)	7.77 ± 1.88	8.85 ± 2.41	ns	
Recovery La^−^ (mmol·L^−1^)	5.70 ± 1.67	7.44 ± 2.15	0.0291*	*d* = −0.90
Recovery ΔLa^−^ (mmol·L^−1^)	2.07 ± 0.75	1.42 ± 0.53	0.0369*	*d* = 1.00

*HR_peak_, highest heart rate; HR_post_, heart rate after recovery; ΔHR, delta heart rate between HR_peak_ and HR_post_; VO_2peak_, highest oxygen uptake; VO_2post_, oxygen uptake after recovery; ΔVO_2_, delta oxygen uptake between VO_2peak_ and VO_2post_; peak La^−^, the highest level of blood lactate; recovery La^−^, blood lactate concentration after recovery; recovery ΔLa^−^, delta blood lactate concentration between peak La^−^ and recovery La^−^*.

^*^ p < 0.05, ^****^ p < 0.0001.

**Figure 3:**
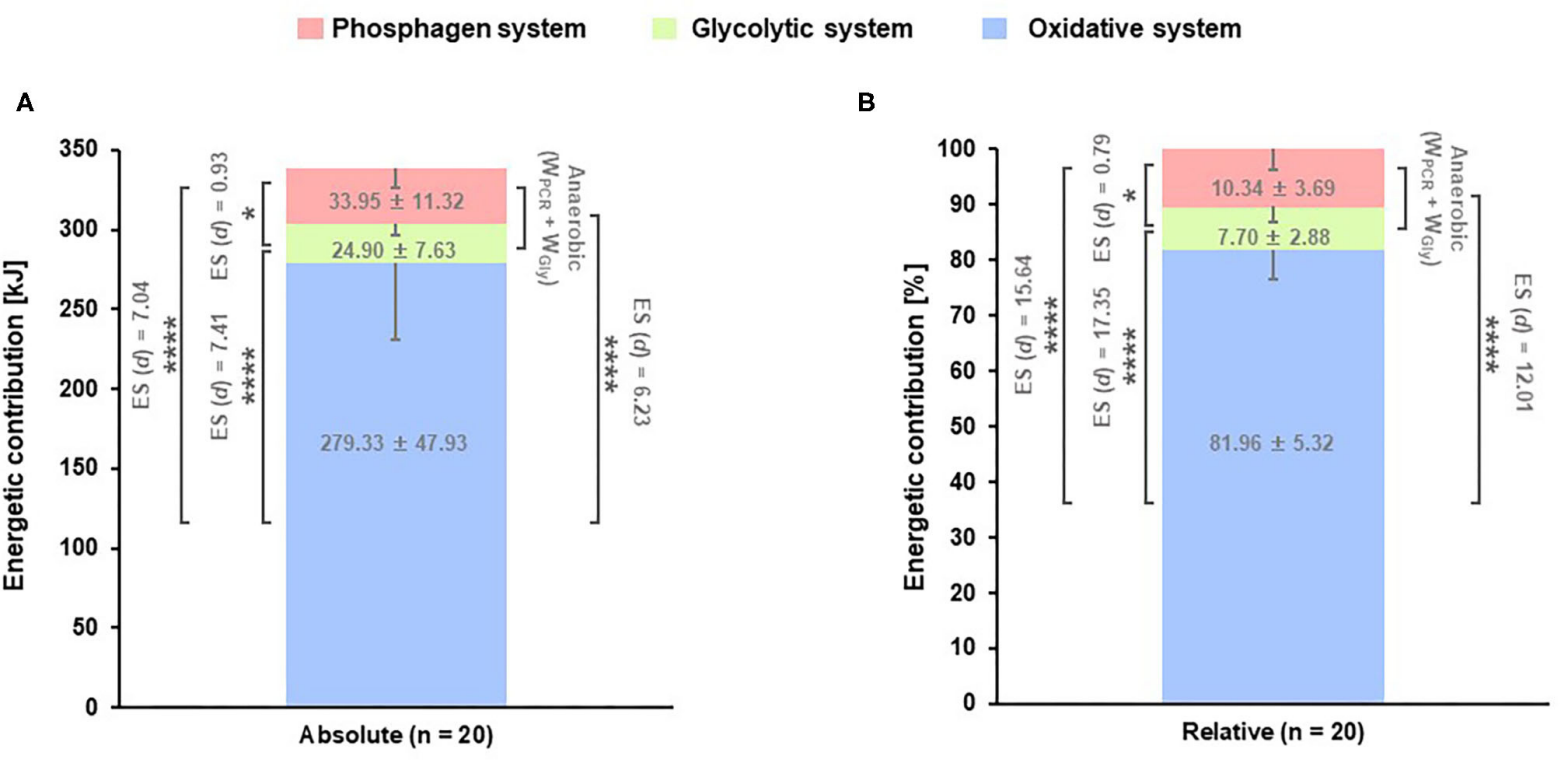
Energetic contribution. *M* ± *SD*. **(A)** Absolute and **(B)** relative energetic contribution during high-intensity hatha yoga. *p < 0.05 (phosphagen vs. glycolytic energy contribution), ****p < 0.0001 (oxidative vs. glycolytic energy contribution), ****p < 0.0001 (oxidative vs. phosphagen energy contribution), ****p < 0.0001 [anaerobic (W_PCR_ + W_Gly_) vs. oxidative energy contribution].

The absolute value (kJ) of W_Oxi_ was significantly higher compared with W_PCR_, W_Gly_, and anaerobic energy contribution (W_PCR_ + W_Gly_) during HIHY [*p* < 0.0001; ES (*d*): 7.04, ES (*d*): −1.30, ES (*d*): 7.41, respectively]. Furthermore, the absolute W_PCR_ value was higher compared with W_Gly_ [*p* = 0.0232; ES (*d*): 0.93] (Figure 3A; Table 2). As well, the relative values (%) for energetic contributions showed the same significant differences as the absolute values [*p* < 0.0001; ES (*d*): 15.64, ES (*d*): −17.35, ES (*d*): 12.01, *p* = 0.0437; ES (*d*): 0.79, respectively] (Figure 3B; Table 2).

### Physiological Parameters During Active and Passive Recovery

**Figure 4:**
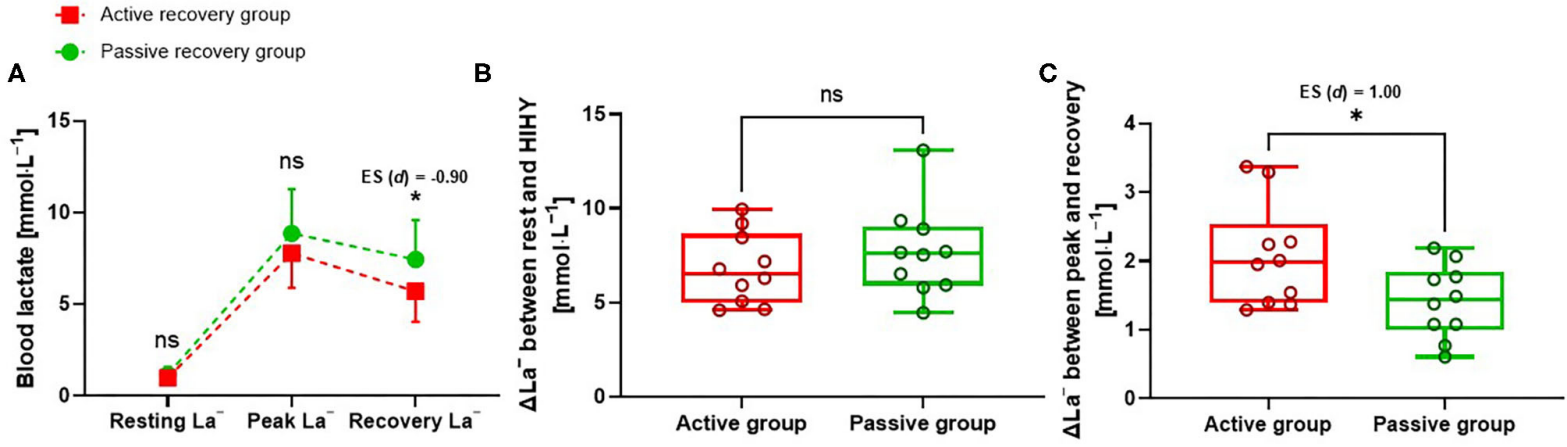
**(A)** Blood lactate concentration for active recovery group and passive recovery group, before high-intensity hatha yoga (HIHY) (resting La^−^), after high-intensity hatha yoga (peak La^−^), after 10-min recovery (recovery La^−^), **(B)** Change in lactate concentration (ΔLa^−^) between resting blood lactate and peak blood lactate after high-intensity hatha yoga, **(C)** Change in lactate concentration (ΔLa^−^) between peak blood lactate after high-intensity hatha yoga and blood lactate after 10-min recovery. Data are minimum to maximum and median values. ns: *p* > 0.05, *p < 0.05.

After different recovery phases, VO_2post_ was significantly higher in the active recovery group compared with the passive group [*p* < 0.0001; ES (*r*): −0.84]. The value of ΔVO_2_ between VO_2peak_ and VO_2post_ was significantly lower in the active group compared with the passive group [*p* = 0.0115; ES (*r*): −0.45] (Table 3). Furthermore, recovery La^−^ was significantly lower in the active group compared with the passive group [*p* = 0.0291; ES (*d*): −0.90] (Figure 4A; Table 3). Accordingly, recovery ΔLa^−^ was significantly higher in the active group compared with the passive group [*p* = 0.0369; ES (*d*): 1] (Figure 4C; Table 3). Other physiological variables such as HR_peak_, HR_post_, ΔHR, VO_2peak_, and peak La^−^ showed no significant differences between groups (Figure 4B; Table 3).

## Discussion

The metabolic energy contributions during HIHY are currently unclear and it is somewhat controversial which recovery methods are physiologically more efficient after HIHY. To the best of our knowledge, this study is the first to evaluate how different energy systems contribute during HIHY and how physiological parameters are influenced by different recovery methods (active vs. passive) afterward. The major findings indicated that oxidative energy predominates over anaerobic energy contributions (W_PCR_ and W_Gly_).

Randomly assigned participants in recovery groups showed no significant differences in HR_peak_, HR_mean_, VO_2peak_, VO_2mean_, and peak La^−^ during HIHY. These indicated that all participants conducted the same HIHY workout. Additionally, values of HR_peak_ (95.6% of HR_max_), HR_mean_ (88.7% of HR_max_), METs (10.5 and 8.5), and peak La^−^ (8.3 mmol·L^−1^) exhibited parameters consistent with HIE (>90%, ≥6 METs; vigorous/heavy, >4 mmol·L^−1^; zone 3: HIE, respectively) (Jetté et al., 1990; Billat, 2001; Ainsworth et al., 2011; Treff et al., 2019; Jamnick et al., 2020). Furthermore, VO_2peak_ (36.9 mL·kg^−1^·min^−1^) indicated a result similar to a previous study in which a VO_2max_ of 37.5 mL·kg^−1^·min^−1^ during HIHY was reported (Table 2) (Papp et al., 2016). Regarding the energetic contribution, a predominant utilization of W_Oxi_ in kJ and % (81.9%) was found and was dominant over W_PCR_, W_Gly_, and the entire anaerobic system (W_PCR_ + W_Gly_). These results were influenced by the duration of HIHY (10 min) and decreased the contribution provided by the glycolytic energy system (Heck et al., 2003; Yang et al., 2020; Park et al., 2021). To obtain a maximal lactate production rate, 10 s exercise duration was suggested because the contribution of the glycolytic system (accumulated lactate rate) decreases with increasing duration of maximal exercise, due to inhibition of phosphofructokinase activity (Heck et al., 2003). Consistent with this, previous studies have shown that the contribution of the oxidative energy system was increased as a consequence of increased VO_2_ uptake during taekwondo (62–70%) and 2,000 m rowing (83–85%), activities which lasted ~6 and 8.5 min, respectively, while the glycolytic system was reduced (de Campos Mello et al., 2009; Campos et al., 2012).

After 10 min recovery phases, the active recovery group (40–45% of HR_max_) had faster lactate clearance (resynthesis) than the passive recovery group (Figures 4A,C). Consequently, higher VO_2post_ and lower ΔVO_2_ were found in the active recovery group compared with the passive (Table 3). This is consistent with a previous study that reported blood lactate concentration after intense running (VO_2max_ and lactate threshold test) was reduced more by active rather than passive recovery regimens with intensities of 25–63% of VO_2max_ and 40–80% of lactate threshold (Menzies et al., 2010). For a low-intensity activity or exercise, such as walking or jogging ATP resynthesis is affected more by substrate-level and oxidative phosphorylation reactions than by accumulated lactate concentration (Rodríguez and Mader, 2011; Yang et al., 2020). In particular, this mechanism is affected by more skeletal muscle activation, including more O_2_ uptake into skeletal muscle cells (Cupeiro et al., 2016; Brooks, 2018; Yang et al., 2020). It mostly occurs in type 1 muscle fibers which predominantly express monocarboxylic transport 1 (MCT1) while MCT2 is prominently expressed in the liver. MCT1 is the most important protein for lactate transport into or out of red blood cells (Menzies et al., 2010; Brooks, 2018; Yang et al., 2020). The study of Yang et al. suggested that accumulated lactate is predominantly eliminated by the Cori cycle during the low-intensity exercise/recovery phase (Yang et al., 2020). As evidence of the mechanism in the liver, hepatic blood flow is increased and muscle lactate output and hepatic lactate uptake are similar during recovery, while a two-third decrease in hepatic blood flow is among the most distinct alterations during HIE in humans (Nielsen et al., 2007). With regard to this aspect, when exercise intensity is higher than 50% of VO_2max_, gluconeogenesis is decreased because of reduced hepatic blood flow (Nielsen et al., 2007; Yang et al., 2020). After active recovery in this study, VO_2post_ was 33% of VO_2peak_ during HIHY (Table 3). Therefore, lactate accumulated during HIHY might be resynthesized in large part by gluconeogenesis during active recovery in the liver. According to the findings of this study, active recovery is more effective at regenerating the metabolic system after 10 min HIHY.

This study identified the energetic contribution during HIHY and physiological differences between active and passive recovery. However, this study has some limitations. The baseline VO_2max_, which can enable the determination of VO_2_ levels during HIHY as percentages of VO_2max_, was not measured in this study. Furthermore, the pure contribution of the calculated glycolytic energy system was limited. This was underestimated because lactate elimination and production rate could not be analyzed between HIHY. Therefore, further studies are expected to investigate different durations of exercise, e.g., 2, 4, 6, 8, and 10 min, which should be randomly determined and blinded to the duration. Furthermore, 10 min HIHY should be used and considered in interventional studies of how cardiovascular/cardiorespiratory fitness such as VO_2max_ and VO_2peak_ will be changed in different populations, such as the unskilled general public.

## Conclusion

Our findings indicated that 10 min HIHY is suitable for an HIE session based on the high levels of physiological parameters and energetic contributions. Because of the high level of exercise intensity, this form of exercise is appropriate for relatively healthy employees in the workplace who may have HY experience, but do not have time for physical exercise. However, for safety, HIHY should be preceded by appropriate warm-up exercises, such as the classical sun salutation, which is performed slowly. Finally, this study found that active recovery is a more helpful method compared with traditional passive (*savasana*) recovery after 10 min HIHY. After active recovery, people can participate in further HIHY sessions during short breaks such as lunchtime. Consequently, a quicker return to the workplace can be supported by metabolic regeneration.

## Data Availability Statement

The original contributions presented in the study are included in the article/supplementary material, further inquiries can be directed to the corresponding author/s.

## Ethics Statement

The studies involving human participants were reviewed and approved by the Institutional Ethics Committee of CHA University (No. 1044308-202007-HR-026-02). The patients/participants provided their written informed consent to participate in this study. Written informed consent was obtained from the individual(s) for the publication of any potentially identifiable images or data included in this article.

## Author Contributions

K-HL and W-HY were involved in study conception and design and wrote the first draft of the manuscript. K-HL, H-MJ, and W-HY collected the data and analyzed the data. All authors revised, edited, and approved the final manuscript.

## Conflict of Interest

The authors declare that the research was conducted in the absence of any commercial or financial relationships that could be construed as a potential conflict of interest.

## Publisher's Note

All claims expressed in this article are solely those of the authors and do not necessarily represent those of their affiliated organizations, or those of the publisher, the editors and the reviewers. Any product that may be evaluated in this article, or claim that may be made by its manufacturer, is not guaranteed or endorsed by the publisher.
